# Neutrophils and Neutrophil-Based Drug Delivery Systems in Anti-Cancer Therapy

**DOI:** 10.3390/cancers17071232

**Published:** 2025-04-05

**Authors:** Hicham Wahnou, Riad El Kebbaj, Soufyane Hba, Zaynab Ouadghiri, Othman El Faqer, Aline Pinon, Bertrand Liagre, Youness Limami, Raphaël Emmanuel Duval

**Affiliations:** 1 Laboratory of Immunology and Biodiversity, Faculty of Sciences Ain Chock, Hassan II University, B.P 2693, Maarif, Casablanca 20100, Morocco; hwwahnou@gmail.com (H.W.); hbasoufyane@gmail.com (S.H.); zaynabouadghiri1@gmail.com (Z.O.); mr.othman.elfaqer@gmail.com (O.E.F.); 2 Sciences and Engineering of Biomedicals, Biophysics and Health Laboratory, Higher Institute of Health Sciences, Hassan First University, Settat 26000, Morocco; elkebbajriad@gmail.com; 3 Univ. Limoges, LABCiS, UR 22722, F-87000 Limoges, France; aline.pinon@unilim.fr (A.P.); bertrand.liagre@unilim.fr (B.L.); 4 Université de Lorraine, F-54000 Nancy, France

**Keywords:** neutrophils, tumor microenvironment, anti-cancer therapy, immunotherapy, neutrophil extracellular traps, drug delivery, inflammation, metastasis

## Abstract

Neutrophils, the most abundant white blood cells, play a dual role in cancer progression. While they can promote tumor growth, metastasis, and immune suppression, they also exhibit anti-tumorigenic properties by attacking cancer cells and enhancing immune responses. This review explores the complex interplay between neutrophils and the tumor microenvironment (TME), highlighting their ability to switch between pro- and anti-tumor phenotypes based on external stimuli. Pro-tumorigenic neutrophils facilitate tumor growth through mechanisms such as neutrophil extracellular traps (NETs), secretion of pro-inflammatory cytokines, and immune evasion strategies. They contribute to angiogenesis, tumor invasion, and metastasis by releasing vascular endothelial growth factor (VEGF) and matrix metalloproteinases (MMPs). Conversely, anti-tumor neutrophils enhance cytotoxicity by generating reactive oxygen species (ROS), promoting antibody-dependent cell-mediated cytotoxicity (ADCC), and activating other immune cells such as cytotoxic T lymphocytes (CTLs) and natural killer (NK) cells. Recent advances in neutrophil-based drug delivery systems have harnessed their tumor-homing capabilities to improve targeted therapy. Neutrophil-mimicking nanoparticles and membrane-coated drug carriers offer enhanced drug accumulation in tumors, reduced systemic toxicity, and improved therapeutic outcomes. Additionally, strategies to modulate neutrophil activity, such as inhibiting their immunosuppressive functions or reprogramming them towards an anti-tumor phenotype, are emerging as promising approaches in cancer immunotherapy. Understanding neutrophil plasticity and their interactions with the TME provides new avenues for therapeutic interventions. Targeting neutrophil-mediated mechanisms could enhance existing cancer treatments and lead to the development of novel immunotherapies, ultimately improving patient survival and clinical outcomes.

## Simple Summary

Neutrophils, a type of immune cell, may play a complex role in cancer and can have completely opposite effects. In fact, while they can help fight tumors by attacking cancer cells, they can also help tumors grow by creating a supportive environment. Scientists are exploring ways to either block neutrophils that help cancer grow, activate the anti-tumorigenic functions of neutrophils or use them to deliver cancer-fighting drugs. This review discusses the different ways neutrophils interact with tumors and highlights new strategies that could turn them into powerful tools for anti-cancer therapy.

## 1. Introduction

Cancer remains one of the leading causes of morbidity and mortality worldwide, characterized by uncontrolled cell proliferation, immune system evasion, and the ability to metastasize to distant organs [1,2]. The complexity of cancer arises from its interactions with the tumor microenvironment (TME), which consists of immune cells, stromal components, and signaling molecules that collectively influence tumor progression [3,4]. Among the various immune cells present in the TME, neutrophils have garnered significant attention due to their paradoxical role in tumor development [5]. In fact, neutrophils are the most abundant white blood cells, with a central role in innate immunity by acting as first responders to infections and tissue damage [6,7]. They rapidly infiltrate affected tissues, eliminating pathogens through phagocytosis, degranulation, and the release of neutrophil extracellular traps (NETs) [8,9,10]. Their interactions with tumor cells and immune components can either stimulate anti-tumor immunity or contribute to cancer progression [11]. While some neutrophils enhance immune responses by releasing cytotoxic molecules and recruiting other immune cells, others facilitate tumor growth by secreting pro-inflammatory cytokines, promoting angiogenesis, and suppressing immune surveillance [12]. Their ability to switch between these opposing roles underscores their significance as a potential therapeutic target [13]. Understanding the mechanisms that govern neutrophil behavior in tumors is essential for developing novel anticancer therapies aimed at modulating their activity for improved patient outcomes.

This review builds upon previous research on tumor-associated neutrophils (TANs) and NETs, expanding our understanding of their dual role in cancer. While neutrophils can enhance anti-tumor immunity by releasing cytotoxic molecules and activating other immune cells, they can also facilitate tumor growth through the secretion of pro-inflammatory cytokines, immunosuppressive factors, and angiogenic mediators [5].

In addition to discussing neutrophil functions, this review highlights the latest therapeutic approaches, including neutrophil-targeted drug delivery systems, immune reprogramming strategies, and inhibitors of neutrophil recruitment and activation. A key focus is the integration of neutrophil-based therapies with conventional cancer treatments to enhance efficacy and overcome resistance.

Furthermore, we address the major challenges, research gaps, and opportunities in this field. Issues such as neutrophil plasticity, tumor specificity, and unintended immunosuppressive effects remain obstacles to clinical translation. By identifying these limitations and proposing innovative solutions, this review aims to guide future research and therapeutic development in neutrophil-targeted oncology. Understanding these complexities may unlock new possibilities for precision medicine and immunotherapy in cancer treatment.

## 2. Neutrophils in Cancer

The dualistic nature of neutrophils, by promoting both tumor growth and anti-tumor activity, makes them a key focus in understanding cancer progression and in developing therapeutic strategies [13]. Neutrophils interact dynamically with the TME, adopting distinct phenotypes based on cues from tumor-derived factors [13]. This section explores their pro-tumorigenic and anti-tumorigenic functions and highlights the concept of neutrophil plasticity.

### 2.1. Pro-Tumorigenic Functions of Neutrophils

Neutrophils can significantly contribute to tumor progression through a range of pro-tumorigenic activities. These functions include promoting tumor cell proliferation, facilitating metastasis, and suppressing anti-tumor immune responses. Key mechanisms include:

#### 2.1.1. Recruitment to the TME

**Figure 1:**
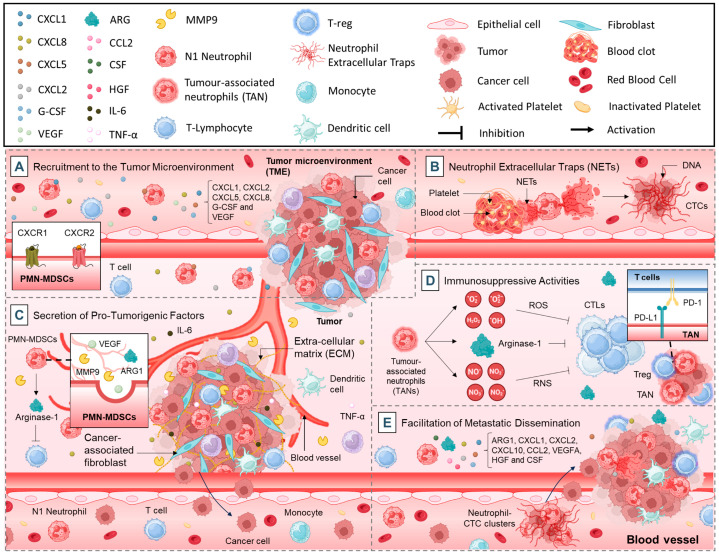
Pro-tumorigenic functions of neutrophils: (**A**). recruitment to the TME, (**B**). Neutrophil Extracellular Traps (NETs), (**C**). secretion of pro-tumorigenic factors, (**D**). immunosuppressive activities, (**E**). facilitation of metastatic dissemination.

Neutrophil recruitment to the TME is a hallmark of their pro-tumorigenic role. Tumor cells and stromal cells secrete a variety of chemokines and cytokines, including cysteine X cysteine ligands such as CXCL1, CXCL2, CXCL5, and CXCL8 (IL-8), that attract neutrophils to the tumor site (Figure 1A) [14]. These signals act via chemokine receptors, such as CXCR1 and CXCR2, on the surface of neutrophils [14,15]. Additionally, factors like granulocyte colony-stimulating factor (G-CSF) and vascular endothelial growth factor (VEGF) contribute to the mobilization and recruitment of neutrophils from the bone marrow [16].

Once recruited, neutrophils accumulate in the TME, where they interact with tumor cells and stromal components [17]. High neutrophil infiltration is frequently associated with poor prognosis in various cancers, including lung, pancreatic, and colorectal cancers [18,19,20,21]. These TANs contribute to tumor growth by enhancing angiogenesis, extracellular matrix remodeling, and immune suppression (Figure 1A) [22,23].

#### 2.1.2. Neutrophil Extracellular Traps (NETs)

Neutrophils can form NETs in response to stimuli from tumor cells and the TME [24]. NETs are web-like structures composed of decondensed chromatin, histones, and granule proteins such as neutrophil elastase and myeloperoxidase [25]. While NETs play a protective role in trapping and killing pathogens, their involvement in cancer is predominantly pro-tumorigenic [26]. In cancer, NETs facilitate metastasis by trapping circulating tumor cells (CTCs) in the vasculature, thereby promoting their adhesion to endothelial cells and extravasation into distant tissues (Figure 1B) [27]. NETs also release proteases and cytokines that degrade the extracellular matrix, creating a permissive environment for tumor invasion [28]. Furthermore, NET-associated components can activate platelets and the coagulation cascade, further supporting metastasis (Figure 1B) [29].

Studies have shown elevated levels of NETs in patients with advanced cancers, and their presence is associated with increased metastatic burden and worse clinical outcomes [30]. Targeting NET formation with agents such as DNase or inhibitors of peptidylarginine deiminase 4 (PAD4), an enzyme critical for NET formation, is being explored as a therapeutic strategy [31].

#### 2.1.3. Secretion of Pro-Tumorigenic Factors

Neutrophils secrete a wide array of factors that promote tumor progression. Among these, VEGF plays a pivotal role by driving angiogenesis, thereby enhancing the blood supply to tumors and ensuring nutrient and oxygen delivery [32]. Another significant group of factors is the matrix metalloproteinases (MMPs), such as MMP-9, which degrade the extracellular matrix to facilitate tumor cell invasion and metastasis (Figure 1C) [33]. In addition to these, neutrophils produce arginase-1 (ARG1), an enzyme that depletes arginine, effectively suppressing T-cell function and fostering an immunosuppressive TME [34]. Pro-inflammatory cytokines, including interleukin-6 (IL-6) and tumor necrosis factor-alpha (TNF-α), further contribute to this microenvironment by creating a feedback loop that enhances tumor cell proliferation and survival (Figure 1C) [35,36]. Collectively, these secreted factors not only support tumor growth but also remodel the TME, aiding immune evasion and metastasis.

#### 2.1.4. Immunosuppressive Activities

TANs play a critical role in suppressing anti-tumor immune responses, thus enabling tumor progression [37]. One of their key mechanisms involves the inhibition of T-cell function through the production of reactive oxygen species (ROS), reactive nitrogen species (RNS), and ARG1, which impair T-cell activation and proliferation by disrupting critical metabolic and signaling pathways (Figure 1D) [38,39]. Moreover, TANs promote the expansion and activity of regulatory T cells (Tregs) by secreting immunosuppressive cytokines such as transforming growth factor-beta (TGF-β) and interleukin-10 (IL-10) [40,41]. This immunosuppressive profile is further compounded by TAN-mediated impairment of dendritic cell maturation and antigen-presenting capabilities, resulting in diminished activation of cytotoxic T lymphocytes (CTLs) (Figure 1D) [42,43]. Additionally, TANs in the TME express immune checkpoint ligands like programmed death-ligand 1 (PD-L1), which interact with (programmed cell death protein 1) PD-1 on T cells to suppress their activity and promote immune evasion (Figure 1D) [44,45].

These mechanisms collectively facilitate immune escape, allowing tumors to thrive and metastasize. Therapeutic strategies that target these immunosuppressive pathways, such as ARG1 inhibitors or PD-L1 blockers, are currently under investigation and offer potential for restoring effective anti-tumor immunity [46].

#### 2.1.5. Facilitation of Metastatic Dissemination

Metastasis is the leading cause of cancer-related mortality, and neutrophils play a pivotal role in this process [47]. During intravasation, neutrophils assist tumor cells in entering the circulation by breaking down the basement membrane and facilitating the detachment of tumor cells from the primary site [48]. Once in the bloodstream, neutrophils interact with CTCs, forming “neutrophil-CTC clusters” that shield the tumor cells from immune attack (Figure 1E) [47]. These clusters also enhance the adhesion of CTCs to endothelial cells, facilitating their extravasation into distant tissues [47].

Neutrophils contribute to the establishment of pre-metastatic niches by secreting chemokines such as ARG1, CXCL1, CXCL2, CXCL10, CCL2, CXCR2, and vascular endothelial growth factor A (VEGFA), which attract tumor cells to metastatic sites (Figure 1E) [47,49]. In these sites, neutrophils release growth factors like hepatocyte growth factor (HGF) and colony-stimulating factor (CSF), which promote tumor cell survival and proliferation [50]. Moreover, neutrophils enhance vascular permeability at the metastatic site, allowing tumor cells to extravasate more easily [51]. By preparing and supporting metastatic niches, neutrophils significantly enhance the ability of cancer cells to colonize distant organs and establish secondary tumors.

### 2.2. Anti-Tumorigenic Functions of Neutrophils

Despite their pro-tumorigenic roles, neutrophils also possess anti-tumorigenic capabilities, particularly in early tumor development or under specific conditions where they adopt an anti-tumor phenotype. Key anti-tumorigenic mechanisms include:

#### 2.2.1. Direct Cytotoxicity

**Figure 2:**
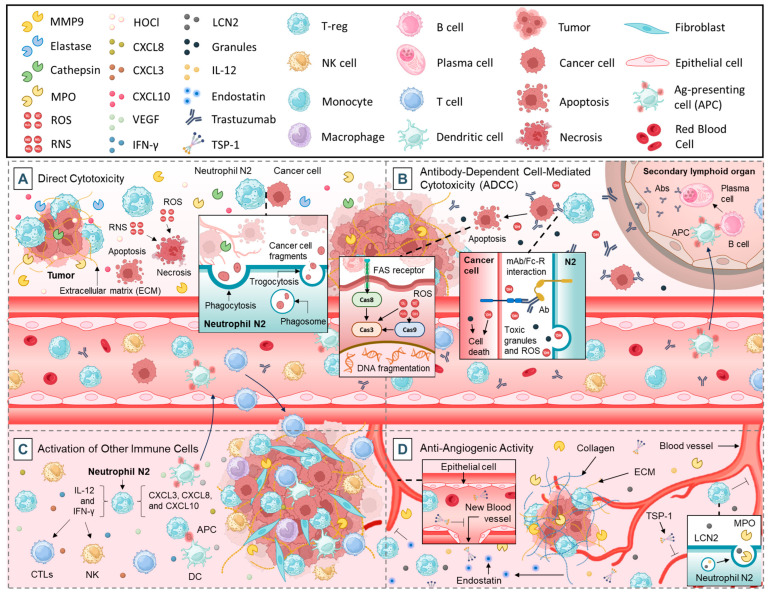
Anti-tumorigenic functions of neutrophils: (**A**). direct cytotoxicity, (**B**). Antibody-Dependent Cell-Mediated Cytotoxicity (ADCC), (**C**)**;** activation of other immune cells, and (**D**). anti-angiogenic activity.

Neutrophils play a pivotal role in tumor cell elimination through a combination of biochemical, enzymatic, and mechanical mechanisms. One of their primary cytotoxic strategies involves the release of ROS and RNS, which induce oxidative stress and damage key cellular components such as DNA, lipids, and proteins (Figure 2A) [12,52]. This damage disrupts the tumor cells’ structural and functional integrity, leading to apoptotic or necrotic cell death [48]. These reactive molecules also impair the tumor’s ability to proliferate and metastasize, making neutrophils key players in antitumor immunity. In addition to ROS and RNS, neutrophils release a range of enzymes that amplify their cytotoxic effects [8]. Proteolytic enzymes such as elastase and cathepsins degrade tumor cell membranes and extracellular matrix (ECM) components, destabilizing the structural support for tumor growth [53]. Myeloperoxidase (MPO) produces hypochlorous acid (HOCl), a highly toxic molecule that damages tumor cell membranes, DNA, and proteins [54]. Gelatinase (MMP-9) further weakens the ECM, facilitating immune cell infiltration and enhancing the delivery of cytotoxic molecules to tumor sites (Figure 2A) [33]. Collectively, these enzymes create a hostile environment for tumor cells, contributing significantly to their destruction.

Neutrophils also utilize mechanical processes like phagocytosis and trogocytosis to eliminate tumor cells [55,56]. During phagocytosis, neutrophils engulf tumor cells or debris, exposing them to a toxic enzymatic cocktail within phagolysosomes, leading to efficient degradation [57]. Trogocytosis, on the other hand, involves the selective stripping of tumor cell membranes, impairing their functionality and exposing them to further immune-mediated attacks (Figure 2A) [58]. Enzymes like elastase and gelatinase enhance these processes by weakening the tumor cell surface, making it more vulnerable to mechanical disruption.

Recent studies suggest that enhancing the cytotoxic capacity of neutrophils through genetic engineering or pharmacological modulation could improve their anti-tumor efficacy, offering potential strategies for novel immunotherapies [59].

#### 2.2.2. Antibody-Dependent Cell-Mediated Cytotoxicity (ADCC)

Neutrophils participate in ADCC, a critical immune mechanism in tumor control [60]. In this process, tumor cells coated with specific antibodies are targeted by neutrophils through the binding of their Fcγ receptors to the Fc region of the antibodies [60], particularly through their FcγRIIIb, which has been associated with ADCC potency [61]. This interaction activates neutrophils, leading to the release of toxic granules, ROS, and inflammatory cytokines that kill tumor cells (Figure 2B) [60,62].

ADCC is particularly relevant in the context of therapeutic monoclonal antibodies, such as trastuzumab (for Human Epidermal Growth Factor Receptor-2 (HER2)-positive breast cancer) and rituximab (for B-cell lymphomas) [63,64]. These treatments are designed to mark tumor cells for destruction by immune cells, including neutrophils. Neutrophil-mediated ADCC has been identified as a key mechanism underlying the success of these therapies [63,64].

Efforts to enhance neutrophil recruitment and activation during monoclonal antibody treatment are ongoing, including the development of next-generation antibodies with increased binding affinity to neutrophil Fcγ receptors [65]. However, IgG Fc engineering has limitations in anti-cancer therapy. Optimizing FcγR binding risks off-target effects like cytokine release or autoimmunity, while immunosuppressive tumor microenvironments may reduce efficacy. FcγR polymorphisms (e.g., FcγRIIIb) also necessitate personalized approaches [66,67]. Despite progress, balancing potency, safety, and tumor heterogeneity remains critical.

#### 2.2.3. Activation of Other Immune Cells

Neutrophils serve as crucial mediators in orchestrating the anti-tumor immune response by activating and recruiting other immune cells [48]. Through the secretion of pro-inflammatory cytokines such as IL-12 and interferon-gamma (IFN-γ), neutrophils promote the activation of CTLs and natural killer (NK) cells, which are directly involved in recognizing and eliminating tumor cells (Figure 2C) [68,69].

Neutrophils also release chemokines, including CXCL3, CXCL8, and CXCL10, which recruit additional immune cells to the tumor site [70]. This recruitment amplifies the overall immune response and fosters a hostile environment for tumor cells (Figure 2C) [70].

Additionally, neutrophils interact with dendritic cells to enhance antigen presentation [71]. By transferring tumor-derived antigens to dendritic cells, neutrophils contribute to the priming of T cells, enabling the adaptive immune system to mount a targeted attack against tumors (Figure 2C) [72,73]. This interplay between neutrophils and other immune cells is critical for initiating and sustaining anti-tumor immunity.

#### 2.2.4. Anti-Angiogenic Activity

Angiogenesis, the formation of new blood vessels, is essential for tumor growth and metastasis [74]. However, under specific conditions, neutrophils can exhibit anti-angiogenic activity, limiting the tumor’s ability to establish a sufficient blood supply [75].

Neutrophils contribute to the suppression of tumor growth and metastasis by releasing anti-angiogenic factors, which inhibit the formation of new blood vessels that tumors rely on for oxygen and nutrient supply. Among these factors, thrombospondin-1 (TSP-1) plays a critical role. TSP-1 is a glycoprotein released by activated neutrophils that binds to endothelial cells, disrupting their proliferation and migration, thereby impeding the angiogenic process (Figure 2D) [76]. Additionally, neutrophils secrete MMPs, such as MMP-9, which, under certain conditions, can liberate anti-angiogenic fragments from ECM components [77]. For instance, the cleavage of collagen produces fragments like endostatin, a potent inhibitor of angiogenesis [78].

Another key anti-angiogenic factor produced by neutrophils is α-defensins, which interfere with VEGF signaling, a primary driver of angiogenesis in tumors [79,80]. By inhibiting VEGF activity, neutrophils reduce endothelial cell proliferation and the formation of vascular networks within the TME (Figure 2D) [81]. Moreover, MPO, an enzyme abundantly expressed in neutrophils, can indirectly exert anti-angiogenic effects by generating ROS that damage endothelial cells and suppress angiogenesis [82]. Furthermore, neutrophil-derived lipocalin-2 (LCN2) was shown to disrupt angiogenesis by modulating iron metabolism and impairing endothelial cell function [83,84].

Through the release of these anti-angiogenic molecules, neutrophils create a hostile microenvironment for tumor growth by depriving the tumor of its vascular support. However, the role of neutrophils in angiogenesis is context-dependent, as they can also exhibit pro-angiogenic properties under certain conditions, emphasizing the complexity of their interactions within the TME [85]. Understanding the mechanisms behind neutrophil-mediated anti-angiogenesis offers potential therapeutic strategies to limit tumor progression.

## 3. Therapeutic Strategies

Neutrophils, particularly polymorphonuclear myeloid-derived suppressor cells (PMN-MDSCs), play a pivotal role in the immunosuppressive TME, hindering the efficacy of cancer therapies [86]. To counteract their deleterious effects, various strategies have been developed to target neutrophils in cancer. These approaches include (i) inhibiting the immunosuppressive functions of PMN-MDSCs, (ii) blocking their recruitment to tumor sites, (iii) disrupting their differentiation, and (iv) directly reducing their numbers. Each of these methods leverages distinct mechanisms, such as targeting immunosuppressive mediators, extracellular structures like NETs, critical signaling pathways, and transcription factors. Additionally, emerging strategies focus on utilizing chemotherapeutic agents and noncoding RNAs to modulate or eliminate PMN-MDSCs. Together, these interventions hold significant promise in enhancing antitumor immunity and improving the outcomes of cancer treatments. This section explores these four complementary strategies, offering insights into their therapeutic potential and mechanisms of action.

### 3.1. Inhibition of the Immunosuppressive Function of PMN-MDSCs

The suppressive activity of PMN-MDSCs is a significant barrier to effective cancer immunotherapy [87]. Recent advancements have identified various therapeutic strategies aimed at reducing the immunosuppressive effects of these cells. These approaches focus on disrupting key signaling pathways, inhibiting transcription factors, targeting secreted immunosuppressive molecules, and neutralizing extracellular structures like NETs. Additionally, noncoding RNAs have emerged as promising targets to modulate PMN-MDSCs activity. This section delves into specific interventions and their potential to enhance the efficacy of cancer treatments.

#### 3.1.1. Targeting Immune Suppressors

**Figure 3:**
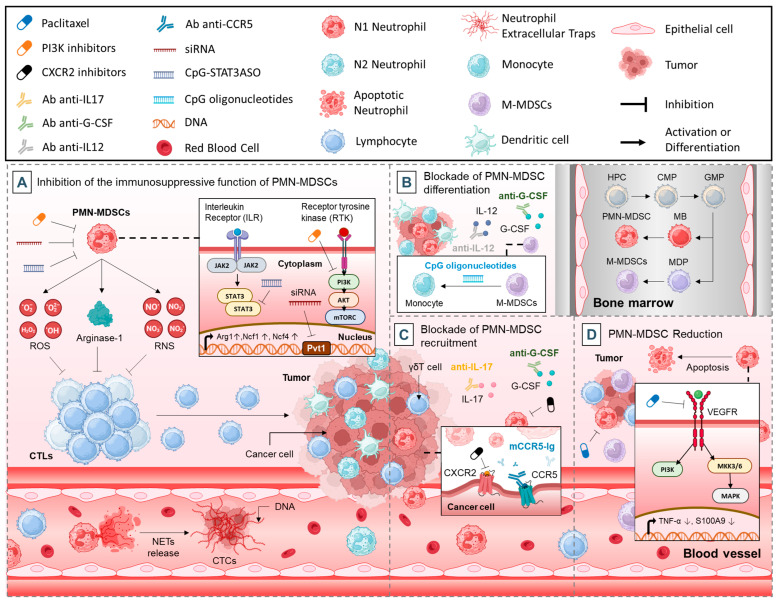
Therapeutic strategies and mechanisms of action, targeting PMN-MDSCs: (**A**). inhibition of the immunosuppressive function of PMN-MDSCs, (**B**). blockade of PMN-MDSCs differentiation, (**C**). blockade of PMN-MDSCs recruitment and (**D**). PMN-MDSCs reduction.

ROS, RNS, and ARG1 are critical mediators of immune suppression. Targeting the immunosuppressive factors secreted by PMN-MDSCs is also an effective strategy (Figure 3A) [88,89,90]. Neutrophil-derived ROS can inhibit T cell proliferation, creating an immunosuppressive environment that is supportive of tumor growth.

RNS, for instance, can disrupt CTL activity by nitrating key proteins such as Tyr394 of lymphocyte-specific protein tyrosine kinase (LCK), thus impairing T cell receptor signaling (Figure 3A) [91]. Neutralizing RNS with agents like uric acid has proven beneficial in reversing these effects and enhancing immunotherapy in both lung and prostate cancer models [91,92]. Similarly, inhibitors of ARG1 activity, such as N-hydroxy-nor-L-arginine (nor-NOHA) and bardoxolone methyl, can alleviate immunosuppression by restoring L-arginine levels crucial for T cell function [88].

#### 3.1.2. Targeting NETs

Another emerging area of research focuses on inhibiting NETs, structures composed of DNA and antimicrobial proteins released during NETosis [93]. While NETs are instrumental in immobilizing pathogens, they also facilitate cancer progression by capturing CTCs, promoting metastasis, and awakening dormant cancer cells (Figure 3A) [25,27]. Inhibitors of NETosis, such as PAD4 inhibitors [94], heparin [95], and Deoxyribonuclease (DNase) I-coated nanoparticles [96] have shown efficacy in preclinical models by reducing metastasis. However, care must be taken with these therapies as NET inhibition might compromise innate immune defenses.

#### 3.1.3. Targeting Tyrosine Kinase Signaling

One approach involves targeting signaling pathways critical for PMN-MDSCs activity. For example, receptor tyrosine kinase signaling can be inhibited using cabozantinib, which, as demonstrated by Patnaik et al., facilitated neutrophil infiltration with antitumor properties into tumors, thereby slowing prostate cancer progression in Pten −/ − p53−/ − mouse models [97]. Furthermore, this drug also diminished the expression of immunosuppressive genes such as Arg1, Ncf1, and Ncf4 in tumor-infiltrating PMN-MDSCs, especially when combined with the dual Phosphoinositide 3-Kinase (PI3K)/mTOR inhibitor dactolisib (Figure 3A) [98]. These agents worked synergistically with immune checkpoint blockade (ICB) therapies to effectively eliminate primary and metastatic prostate tumors in genetically engineered mouse models [98]. Isoform-selective PI3K inhibitors, including PI3Kγ inhibitors [99], PI3Kβ inhibitors [100], and PI3Kδ/γ inhibitors [99], have also been effective in suppressing tumor-promoting myeloid cells and enhancing immunotherapy outcomes.

#### 3.1.4. Targeting STAT3

Silencing key transcription factors such as signal transducer and activator of transcription 3 (STAT3) offers another therapeutic avenue. STAT3 inhibition via antisense oligonucleotides tethered to CpG oligonucleotides (CpG-STAT3ASO) has reduced circulating PMN-MDSCs and improved the CTL-to-Treg ratio in prostate cancer models (Figure 3A) [101]. Similarly, cyclooxygenase-2 inhibitors like celecoxib and SC-236 have shown promise in reducing PMN-MDSCs numbers and activity by suppressing STAT3 in myeloid cells [102,103,104,105,106]. Another molecule of interest, S100A8/A9, which is involved in chronic and acute inflammation via Toll-like receptor 4 (TLR4) or receptor for advanced glycation endproducts (RAGE)-mediated pathways, was targeted with peptide-Fc fusion proteins [107]. These agents effectively eliminated granulocytic and monocytic MDSCs in mice, outperforming anti-Gr1 antibodies [108].

#### 3.1.5. Targeting Noncoding RNAs

Noncoding RNAs like Pvt1 have emerged as regulators of PMN-MDSCs immunosuppressive activity [109]. Silencing Pvt1 using siRNA significantly impaired the suppressive functions of PMN-MDSCs in a Lewis lung carcinoma mouse model, offering a novel target for therapeutic intervention (Figure 3A) [87].

### 3.2. Blockade of PMN-MDSCs Differentiation

Another approach focuses on disrupting the generation and differentiation of PMN-MDSCs. Tumor-derived cytokines like granulocyte-macrophage colony-stimulating factor (GM-CSF), G-CSF, and VEGF promote the accumulation of immature myeloid cells while inhibiting their maturation into functional myeloid cells such as macrophages, dendritic cells, and granulocytes [110,111]. Therapeutics that block these cytokines, their receptors, or downstream pathways were shown to reduce PMN-MDSCs populations. For instance, IL-12, anti-G-CSF antibodies, and all-trans retinoic acid (ATRA) have demonstrated success in decreasing PMN-MDSCs numbers and activity (Figure 3B) [112].

An alternative tactic involves redirecting PMN-MDSCs towards differentiation into mature myeloid cells with diminished immunosuppressive functions [113]. Studies have reported that ultra-low doses of paclitaxel, which do not induce PMN-MDSCs apoptosis, promote their differentiation into dendritic cells in a TLR4-independent manner [114]. Similarly, intratumoral administration of CpG oligonucleotides was shown to convert MDSCs into macrophages with enhanced tumoricidal properties (Figure 3B) [113].

### 3.3. Blockade of PMN-MDSCs Recruitment

A promising approach to mitigating the immunosuppressive effects of PMN-MDSCs is to inhibit their recruitment to the TME. Specific chemokine signaling pathways, such as those mediated by CXCR2 and CCR5, play a central role in directing PMN-MDSCs to tumors [115]. Studies have demonstrated that CXCR2 inhibitors, including SB225002 and SX-682, and neutralizing antibodies can effectively block PMN-MDSCs infiltration, thereby slowing tumor progression and restoring responsiveness to immunotherapy in refractory cancer models (Figure 3C) [116,117,118]. Similarly, blocking CCR5 signaling with fusion proteins like mCCR5-Ig has shown efficacy in reducing PMN-MDSCs accumulation in melanoma models [119]. In breast cancer metastasis models, targeting the γδT cell/IL-17/neutrophil axis by neutralizing IL-17 or G-CSF suppressed neutrophil recruitment and reversed the immunosuppressive phenotype of T cells (Figure 3C) [112].

### 3.4. PMN-MDSCs Reduction

Certain chemotherapeutic drugs have been found to significantly impact the viability of PMN-MDSCs. Gemcitabine (a deoxycytidine analog that inhibits ribonucleotide reductase), a chemotherapy commonly used against various cancers, including pancreatic cancer, is known to induce myelotoxicity as one of its side effects [120]. In a study by Eriksson et al., gemcitabine was shown to reduce circulating PMN-MDSCs and TGFβ-1 levels while increasing the ratio of effector T cells to Tregs in patients with pancreatic adenocarcinoma [121]. The results indicated a significant reduction in granulocytic PMN-MDSCs in peripheral blood, eight days post-treatment, while monocytic MDSCs remained unaffected, suggesting that gemcitabine can effectively deplete PMN-MDSCs and potentially improve the efficacy of immunotherapy [121].

Furthermore, Sevko et al. demonstrated that ultra-low, non-cytotoxic doses of paclitaxel, a chemotherapeutic drug derived from the bark of the Pacific yew tree (*Taxus brevifolia*) [122], significantly impact the functions of MDSCs, chronic inflammatory mediators, and T cell activities within the TME in vivo [123]. Administration of paclitaxel resulted in a marked decrease in the accumulation and immunosuppressive activities of tumor-infiltrating MDSCs, with no detectable alterations in bone marrow hematopoiesis [123]. This effect was mechanistically associated with the inhibition of p38 MAPK activity, decreased TNF-α production, and reduced S100A9 expression in MDSCs (Figure 3D) [123]. Moreover, the production of mediators driving chronic inflammation within the tumor microenvironment was notably diminished. Importantly, these effects culminated in reduced tumor burden and improved animal survival, outcomes largely mediated by the restoration of CD8+ T cell effector functions [123].

Another chemotherapeutic agent, 5-fluorouracil (5-FU), is an antimetabolite agent that targets thymidylate synthase and works by inhibiting DNA and RNA synthesis, leading to cell death [124]. Research by Vincent et al. demonstrated that 5-FU decreased the number of MDSCs in the TME by inducing apoptosis and promoting IFN-γ production by T cells infiltrating the tumor, thereby boosting antitumor immune responses in a mouse model (Figure 3D) [125]. Importantly, 5-FU did not preferentially target monocytic or granulocytic MDSCs, and it had minimal effects on other immune cell populations such as T cells, NK cells, dendritic cells, or B cells. Thus, both gemcitabine and 5-FU hold promise as agents for targeting and eliminating PMN-MDSCs, with their potential antitumor effects likely linked to their selective depletion of these cells [125].

## 4. Clinical Implications

Clinical trials of targeted neutrophil therapies for cancers aim to evaluate the safety, efficacy, and biological impact of novel interventions targeting key molecular pathways such as CXCR1/CXCR2, STAT3, IL-6, TGF-β, and Cluster of Differentiation 47—Signal Regulatory Protein Alpha (CD47-SIRPα). These pathways play critical roles in tumor progression, immune modulation, and resistance to conventional treatments. Table 1 summarizes the key interventions, their targets, trial phases, and outcomes across a range of cancers, including breast, pancreatic, prostate, colorectal, and more. It highlights significant findings, such as neutropenia management, progression-free survival, safety profiles, and pharmacokinetics, offering a detailed landscape of therapeutic advancements in this field.

## 5. Neutrophil-Based Drug Delivery Systems

Neutrophils, as key players in the immune system, have garnered increasing attention for their potential use as drug delivery vehicles in anti-cancer therapy [133]. These white blood cells are adept at infiltrating tumors, interacting with both immune and stromal cells, and navigating complex microenvironments. Their natural abilities to target and modulate immune responses make them promising candidates for advancing drug delivery strategies aimed at improving therapeutic outcomes [133]. In recent years, neutrophil-based platforms have been developed to (i) enhance tumor targeting, (ii) modulate the immune microenvironment, (iii) induce apoptosis in cancer cells, and (iv) improve drug accumulation at tumor sites (Table 2). This section explores the various ways neutrophil-mimetic systems have been harnessed for drug delivery, focusing on their therapeutic effects, the biological mechanisms they engage, and their potential to revolutionize cancer treatment approaches.

### 5.1. Neutrophil Membrane-Mimetics as Drug Delivery Vehicles

**Figure 4:**
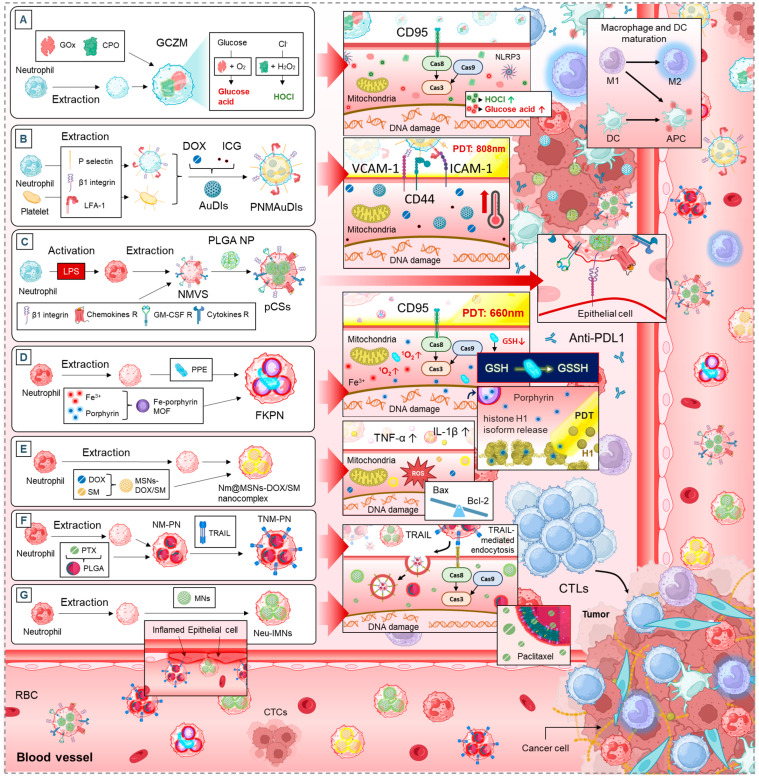
Synthesis of neutrophil membrane-mimetic drug delivery systems and their mechanisms of action: (**A**). artificial “super neutrophils” (GCZM), (**B**). platelet–neutrophil hybrid membrane-coated gold nanocages (PNMAuDIs), (**C**). pseudoneutrophil cytokine sponges (pCSs), (**D**). neutrophil-mimicking nanodevice (FKPN), (**E**). Nm@MSNs-DOX/SM nanocomplex, (**F**). neutrophil membrane-coated nanoparticles (TNM-PN), (**G**). neutrophil membrane-coated immunomagnetic nanoparticles (Neu-IMNs).

Neutrophils, as essential components of the immune system, have been exploited in recent years as vectors for targeted drug delivery in anti-cancer therapy [5]. Their ability to infiltrate tumors and interact with various immune and stromal cells makes them promising candidates for advanced drug delivery strategies [5]. This section highlights different neutrophil-based platforms, their therapeutic effects, and the common biological mechanisms they engage (Figure 4).

#### 5.1.1. Apoptosis and Tumor Growth Suppression

Several drug delivery systems leverage neutrophils to enhance apoptosis in cancer cells. The artificial “super neutrophils” (GCZM) system increases DNA damage, mitochondrial dysfunction, and apoptosis, ultimately reducing tumor growth and metastases (Figure 4A) [134]. Similarly, the supramolecular core–shell nanogel system (SCNG) triggers apoptosis by increasing ROS levels, impairing the G0/G1 checkpoint, and reducing tumor proliferation [135].

The neutrophil-mimicking nanodevice (FKPN) adopts a unique approach by generating singlet oxygen (in situ), leading to DNA damage, histone H1 translocation, and apoptosis while also inducing an abscopal effect, a phenomenon in which localized therapy enhances systemic anti-tumor immunity (Figure 4D) [139].

These strategies are interconnected through their shared mechanisms: induction of apoptosis via mitochondrial dysfunction and ROS generation, as well as direct DNA damage, which enhances the overall cytotoxic effect on tumor cells.

#### 5.1.2. TME Modulation and Immune Activation

In addition to direct tumor cell apoptosis, some systems target the immunosuppressive TME. The hybrid cellular membrane nanovesicles (hNVs) system acts by blocking CD47-SIRPα signaling, enhancing macrophage phagocytosis, and promoting M2-to-M1 macrophage repolarization. This shifts the immune landscape towards a pro-inflammatory, tumor-fighting state [136]. Similarly, pseudoneutrophil cytokine sponges (pCSs) prevent myeloid-derived suppressor cell (MDSC) expansion and tumor infiltration, which enhances T cell function and synergizes with PD-1 blockade therapy (Figure 4C) [138].

Likewise, neutrophil membrane-coated nanoparticles (TNM-PN) contribute to immune activation by promoting tumor-infiltrating T cells and increasing survival rates in tumor-bearing mice (Figure 4F) [141]. These strategies all converge on the idea of transforming the TME from an immune-suppressive state to an immune-reactive one, boosting both innate and adaptive anti-cancer responses.

#### 5.1.3. Tumor Targeting and Drug Accumulation

Neutrophil-based systems also aim to improve drug delivery by enhancing tumor penetration and accumulation. The platelet–neutrophil hybrid membrane-coated gold nanocages (PNMAuDIs) system enhances cellular uptake and tumor accumulation, leading to better therapeutic outcomes (Figure 4B) [137]. A similar concept is employed by neutrophil membrane-coated poly(lactic-co-glycolic acid) (PLGA) nanoparticles (NM-PN) and neutrophil membrane-coated immunomagnetic nanoparticles (Neu-IMNs), which enhance adhesion to inflamed endothelial cells and increase circulation time (Figure 4F,G) [141,144].

Another noteworthy example is the neutrophil membrane-camouflaging nanoparticles (TNM-PN) system, which uses TRAIL-mediated endocytosis to ensure targeted drug uptake. This approach maximizes the therapeutic effects while minimizing off-target toxicity (Figure 4F) [143].

#### 5.1.4. Synergistic Effects with Standard Cancer Therapies

Several of these strategies show potential in combination with existing treatments. The pCSs system, for instance, demonstrates synergy with PD-1 blockade therapy by enhancing the recruitment of anti-tumor T cells (Figure 4C) [138]. Similarly, Nm@MSNs-DOX/SM nanocomplex enhances the effects of conventional chemotherapeutics such as doxorubicin (DOX), through mitochondrial dysfunction and apoptosis induction (Figure 4E) [140].

This synergy suggests that neutrophil-based systems could serve as powerful adjuvants in current cancer treatment regimens, improving efficacy and reducing resistance to standard therapies.

### 5.2. Neutrophils as Multifunctional Delivery Platforms for Multiple Therapies

This section provides an overview of systems using neutrophils as carriers for chemotherapy, immunotherapy, and photodynamic therapy (PDT), each leveraging unique biological mechanisms to enhance therapeutic efficacy.

#### 5.2.1. Enhancing Tumor Targeting and Accumulation

**Figure 5:**
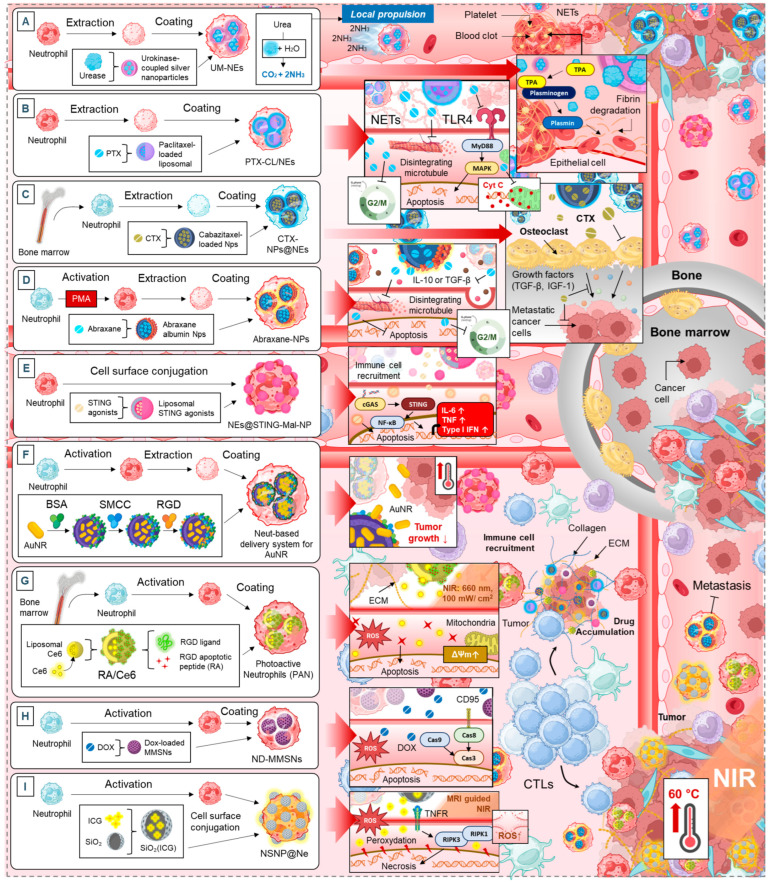
Synthesis of neutrophil membrane-mimetic drug delivery systems and their mechanisms of action: (**A**). urease micromotor-powered neutrophils (UM-NEs) nanodrug delivery system, (**B**). paclitaxel-loaded liposomal neutrophils (PTX-CL/NEs), (**C**). CTX-NPs@NEs (cabazitaxel-loaded nanoparticles with neutrophils), (**D**). abraxane/human neutrophils cytopharmaceuticals, (**E**). neotype neutrophil cytopharmaceutical (NEs@STING-Mal-NP) with liposomal STING agonists, (**F**). neutrophil-based delivery system for Au nanorods (AuNR), (**G**). photoactive neutrophils (PAN) encapsulated multifunctional nanocomplex (RA/Ce6) of RGD apoptotic peptide conjugate (RA) decorated liposomal photosensitizer Ce6, (**H**). inflammation-activatable engineered neutrophils: Neutrophil/Dox-loaded MMSNs (ND-MMSNs), (**I**). nanoparticle–neutrophil composites (NSNP@Ne).

One of the major advantages of neutrophil-based drug delivery is the ability to improve tumor targeting while reducing systemic toxicity. Several systems have been developed to enhance drug accumulation at tumor sites. Paclitaxel-loaded liposomal neutrophils (PTX-CL/NEs) significantly increase drug accumulation in brain tumors, achieving an AUCbrain over 1162 times higher than conventional taxol (Figure 5B) [146]. Similarly, neutrophil/Dox-loaded mesoporous silica nanoparticles (ND-MMSNs) improve drug delivery to glioma sites, leading to increased cytotoxicity and enhanced survival (Figure 5H) [154]. The neutrophil membrane-coated immunomagnetic nanoparticles (Neu-IMNs) system focuses on isolating CTCs, improving early detection and targeted therapy [154]. Additionally, the neutrophil-based delivery system for Au nanorods (AuNR) and photoactive neutrophils (PAN) encapsulated nanocomplex (RA/Ce6) utilize neutrophils to achieve better tumor penetration, delivering photothermal and photodynamic therapies with high precision (Figure 5F,G) [152,153]. These strategies demonstrate that neutrophil-mediated drug delivery enhances accumulation at tumor sites, maximizing therapeutic effects while minimizing off-target toxicity.

#### 5.2.2. Apoptosis Induction and Photothermal Therapy

Many neutrophil-based systems trigger apoptosis through oxidative stress, mitochondrial disruption, and enhanced cytotoxicity. The PAN-encapsulated nanocomplex (RA/Ce6) induces mitochondrial membrane potential disruption and ROS generation, leading to selective apoptosis in cancer cells while sparing healthy tissue (Figure 5G) [153]. Similarly, neutrophil-based delivery of AuNR exploits the photothermal effect to increase tumor temperatures up to 60 °C under near-infrared irradiation, causing cancer cell necrosis and reducing tumor recurrence (Figure 5F) [152]. Pyropheophorbide-a loaded albumin nanoparticles (Ppa-loaded BSA NPs) enhance PDT by increasing neutrophil recruitment to tumor sites, improving drug accumulation, and suppressing tumor growth [151]. These approaches leverage neutrophils not only as carriers but also as active participants in cancer cell destruction, making them highly effective in photothermal and photodynamic applications.

#### 5.2.3. Neutrophils to Reprogram the Tumor Microenvironment

Beyond direct tumor killing, neutrophil-based systems play a crucial role in modulating the immune response within tumors. Neotype neutrophil cytopharmaceuticals (NEs@STING-Mal-NP) activate the stimulator of interferon genes (STING) pathway, promoting macrophage and dendritic cell infiltration and boosting CD8+ T cell-mediated anti-tumor responses (Figure 5E) [150]. Abraxane/human neutrophils cytopharmaceuticals stimulate NETs formation, increasing inflammatory cytokine release (e.g., IL-8, TNF-α), which enhances immune activation against tumors (Figure 5D) [149]. Sialic acid-modified liposomal epirubicin (EPI-SL) depletes tumor-associated macrophages, reducing their immunosuppressive effects and allowing for more effective anti-tumor activity [147]. These findings suggest that neutrophil-based drug delivery can reshape the immune microenvironment, making tumors more susceptible to immune attack and improving the overall efficacy of cancer immunotherapies.

#### 5.2.4. Metastasis and Bone-Associated Cancers

Neutrophil-mediated delivery systems have shown great potential in targeting metastatic cancers, particularly those affecting the bone and vascular systems. CTX-NPs@NEs (cabazitaxel-loaded nanoparticles with neutrophils) effectively inhibit bone metastases in breast cancer models, leading to improved survival rates and preserved bone mineral density (Figure 5C) [148]. This system also reduces splenomegaly, indicating a decrease in systemic inflammatory burden [148]. Urease micromotor-powered neutrophils (UM-NEs) address thrombus-associated complications by restoring vascular recanalization while reducing hemorrhagic side effects and preventing rethrombosis (Figure 5A) [145]. These strategies highlight the versatility of neutrophils in targeting not only primary tumors but also metastatic niches and tumor-associated vascular abnormalities, expanding their potential clinical applications.

## 6. Challenges, Research Gaps and Opportunities

The clinical translation of neutrophil-based delivery platforms faces several hurdles. First, achieving specific and efficient drug delivery to tumors remains a challenge. While systems like PTX-CL/NEs and ND-MMSNs enhance drug accumulation, their efficacy varies across cancer types due to heterogeneous tumor vascularization and stromal barriers. Second, the short lifespan of neutrophils limits their ability to sustain therapeutic payload release, necessitating frequent dosing or engineering solutions to prolong activity. Third, unintended immune consequences, such as systemic inflammation or immunosuppression, may arise from therapies like AuNR and PAN, which rely on oxidative stress or photothermal effects (Figure 5F,G). Finally, the complexity of the TME, including immunosuppressive factors, hypoxia, and dense extracellular matrices, hinders neutrophil infiltration and function, particularly in metastatic niches targeted by UM-NEs and CTX-NPs@NEs (Figure 5A,C).

Key gaps hinder the optimization of neutrophil-based platforms. The mechanisms driving neutrophil homing to tumors are poorly understood, limiting strategies to enhance their tumor-targeting precision. Additionally, the interplay between engineered neutrophils and immune cells (e.g., T cells, macrophages) is understudied. There is also a lack of standardized protocols for scaling up neutrophil engineering, which is critical for reproducibility and clinical adoption. Furthermore, the role of neutrophils in disrupting metastatic niches, such as their ability to penetrate vascular abnormalities or dormant TME, requires deeper mechanistic insights to refine therapies like UM-NEs (Figure 5A).

Advanced bioengineering tools, such as Clustered Regularly Interspaced Short Palindromic Repeats (CRISPR)-based genetic modification or synthetic biology, could extend neutrophil lifespan, enhance tumor homing, and enable controlled payload release. Combination therapies integrating neutrophil platforms with immune checkpoint inhibitors or targeted drugs may overcome microenvironmental resistance and amplify efficacy, as seen in preclinical models combining EPI-SL with PD-1 blockade. Real-time imaging technologies, like neutrophil-tracking nanoparticles or intravital microscopy, could provide dynamic insights into drug delivery efficiency and neutrophil behavior in vivo. Additionally, leveraging neutrophils for early intervention in metastasis by targeting premetastatic niches or circulating tumor cells could prevent advanced disease progression. Finally, patient-specific neutrophil engineering, guided by biomarkers or omics profiling, may enable personalized therapies tailored to tumor biology and immune context. By bridging these gaps, neutrophil-based platforms could revolutionize oncology, offering precise, multifunctional, and minimally invasive treatments for even the most aggressive cancers.

## 7. Conclusions

Neutrophils play a dual role in cancer, acting as both tumor promoters and immune defenders. Their plasticity within the TME presents both challenges and opportunities for therapeutic intervention. Emerging strategies, including neutrophil-targeted drug delivery systems and immune reprogramming, offer promising avenues for improving cancer treatment. However, key challenges such as neutrophil heterogeneity, tumor specificity, and unintended immunosuppressive effects must be addressed. By advancing our understanding of neutrophil biology and refining therapeutic approaches, we can unlock new possibilities for precision oncology, ultimately enhancing treatment efficacy and improving patient outcomes in the fight against cancer.

## Figures and Tables

**Table 1:** Clinical trials of targeted neutrophil therapies for cancers (https://clinicaltrials.gov) accessed: 19 January 2025.

Breast Cancer				
Intervention/Treatment	Target	Phase	Identifier/ References	Results
Efbemalenograstim alfa (F-627)	G-CSF	III	NCT04174599 [126]	Shorter and less severe neutropenia in cycles 2–4; ANC Higher with F-627 across all cycles ↑; Tolerance ↑.
Reparixin (R) + paclitaxel (PTX)	CXCR1/CXCR2	II	NCT02370238 [127]	PFS (↔): Median PFS similar for R + P (5.5 months) and placebo + P (5.6 months); CSC Markers (↓): ALDH+ in 16/54, CD24−/CD44+ in 34/54 metastatic biopsies; Safety ↔
**Pancreatic Cancer**				
**Intervention/Treatment**	**Target**	**Phase**	**Identifier**	**Results**
AZD9150 + MEDI4736	STAT3	II	NCT02983578	No Results Posted.
NIS793 + Nab-paclitaxel/gemcitabine	TGF-β	III	NCT04935359	No Results Posted.
SX-682 + tislelizumab	CXCR1/CXCR2	II	NCT05604560	Recruiting.
**Prostate Cancer**				
**Intervention/Treatment**	**Target**	**Phase**	**Identifier/** **References**	**Results**
AZD5069 + enzalutamide (ENZA)	CXCR1/CXCR2	I/II	NCT03177187 [128]	AZD5069 dose escalated from 160 mg Bruceine D (BD) to 320 mg BD due to ENZA-induced CYP3A4 metabolism; Safety ↔; Efficacy ↑.
**Hepatocellular Carcinoma**				
**Intervention/Treatment**	**Target**	**Phase**	**Identifier**	**Results**
BMS-986253 + nivolumab	IL-8	II	NCT04050462	No Results Posted.
**Colorectal Cancer**				
**Intervention/Treatment**	**Target**	**Phase**	**Identifier/** **References**	**Results**
AZD9150 + MEDI4736	STAT3	II	NCT02983578	No Results Posted.
BBI-608 + FOLFIRI (5-FU, leucovorin, irinotecan)		III	NCT02753127 [129]	Neutrophil count ↓; Safety ↔
**Head and Neck Cancer**				
**Intervention/Treatment**	**Target**	**Phase**	**Identifier**	**Results**
TTI-101 + pembrolizumab	STAT3	I/II	NCT05668949	No Results Posted.
**Melanoma**				
**Intervention/Treatment**	**Target**	**Phase**	**Identifier**	**Results**
Tocilizumab + ipilimumab and nivolumab	IL-6	II	NCT03999749	No Results Posted.
**Non-Hodgkin’s Lymphoma**				
**Intervention/Treatment**	**Target**	**Phase**	**Identifier/** **References**	**Results**
CC-95251 + rituximab	CD47-SIRPα	I	NCT03934814 [129]	No dose-limiting toxicity (DLT) observed up to 30 mg/kg; Average Hgb during the first cycle ↓; Linear PK at mid- to high-dose levels after a single dose; Complete CD47 receptor saturation on peripheral T cells at 20 mg/kg and above.
Lemzoparlimab + rituximab		I	NCT03783403	No Results Posted.
**Multiple Myeloma**				
**Intervention/Treatment**	**Target**	**Phase**	**Identifier**	**Results**
Siltuximab	IL-6	II	NCT01484275	No Results Posted.
**Rectal Cancer**				
**Intervention/Treatment**	**Target**	**Phase**	**Identifier/** **References**	**Results**
LY2157299 + neoadjuvant chemoradiation	TGF-β	II	NCT02688712 [130]	The complete response rate ↑ to 32%; Tolerance ↑.
**Solid Tumors**				
**Intervention/Treatment**	**Target**	**Phase**	**Identifier/** **References**	**Results**
TJ210001	C5aR	I	NCT04947033	No Results Posted.
IPH5401 + durvalumab		I	NCT03665129 [131]	Minimal anti-tumor activity; Tolerance ↑.
Navarixin + pembrolizumab	CXCR1/CXCR2	II	NCT03473925 [132]	No sufficient efficacy in advanced/metastatic CRPC, MSS CRC, or NSCLC; ANC **↓**; Tolerance ↑.
M7824 (bintrafusp alfa)	TGF-β	III	NCT03631706	No Results Posted.
BI 765063 + BI 754091	CD47-SIRPα	I	NCT03990233	No Results Posted.
**Acute Myeloid Leukemia**				
**Intervention/Treatment**	**Target**	**Phase**	**Identifier**	**Results**
Magrolimab + azacitidine	CD47-SIRPα	III	NCT04778397	No Results Posted.

**↑**: Increase; **↓**: Decrease; **↔**: Stable. 5-FU: 5-Fluorouracil; ALDH+: Aldehyde Dehydrogenase-positive; ANC: Absolute Neutrophil Count; BD: Twice Daily; C5aR: Complement Component 5a Receptor; CD24-/CD44+: Cluster of Differentiation 24-negative/CD44-positive; CD47-SIRPα: Cluster of Differentiation 47-Signal Regulatory Protein Alpha; CSC: Cancer Stem Cell; CRPC: Castration-Resistant Prostate Cancer; CXCR1/CXCR2: C-X-C Chemokine Receptor Type 1/Type 2; DLT: Dose-Limiting Toxicity; ENZA: Enzalutamide; FOLFIRI: Folinic Acid, Fluorouracil, and Irinotecan; G-CSF: Granulocyte Colony-Stimulating Factor; Hgb: Hemoglobin; IL-6: Interleukin-6; IL-8: Interleukin-8; MSS CRC: Microsatellite Stable Colorectal Cancer; NSCLC: Non-Small Cell Lung Cancer; PFS: Progression-Free Survival; PK: Pharmacokinetics; PTX: Paclitaxel; R: Reparixin; STAT3: Signal Transducer and Activator of Transcription 3; TGF-β: Transforming Growth Factor Beta.

**Table 2:** Neutrophil-based drug delivery systems for anti-cancer therapy.

Technology	Model	Concentration	Mechanisms	Reference
Artificial “super neutrophils” (GCZM) (Figure 4A)	in vitro: 4T1 and 3T3 cells in vivo: 4T1-xenograft in mice	in vitro: 0.41–4.11 µg GOx/mL and 0.21–2.07 mg CPO/mL in vivo (iv): 0.62 mg GOx/kg and 0.31 mg CPO/kg	DNA damage ↑ Mitochondrial dysfunction ↑ Apoptosis ↑ Necrosis ↑ Tumor growth ↓ Metastatic nodules ↓ Tumor accumulation ↑ GSH ↓	[134]
Supramolecular core–shell nanogel system (SCNG)	in vitro: HepG2 and HL-7702 cells in vivo: HepG2-xenograft in mice	in vitro: IC_50_: 291.24 µg/mL in vivo (iv): 20 mg/kg	ROS levels ↑ Apoptosis ↑ DNA damage ↑ G0/G1 checkpoint ↓ Proliferation ↓ Tumor volume ↓ ^1^O_2_ levels ↑ HOCl levels ↑	[135]
Hybrid cellular membrane nanovesicles (hNVs)	in vitro: B16F10 and 4T1 cells in vivo: B16F10 and 4T1-xenograft in mice	in vitro: 10–500 µg/mL in vivo (iv): 300 µg hNVs/mouse (B16F10), 300 µg hNVs + 36 µg cGAMP/mouse (4T1)	CD47-SIRPα blockade ↑ Macrophage phagocytosis ↑ M2-to-M1 repolarization ↑ Tumor-infiltrating T cells (CD8+) ↑ Tumor recurrence ↓ Metastasis ↓ Survival rate ↑ Cytokine levels (IFN-γ, TNF-α, IL-12) ↑ CTC interaction ↑	[136]
Platelet–neutrophil hybrid membrane-coated gold nanocages (PNMAuDIs) (Figure 4B)	in vitro: 4T1 and MDA-MB-231 cells in vivo: 4T1-xenograft and orthotopic breast tumor-bearing mice	in vitro: 2.5 µg/mL DOX and ICG in vivo (iv): 2.5 mg/kg DOX and ICG	Cellular uptake ↑ Tumor penetration ↑ Cytotoxicity ↑ Tumor growth ↓ Metastatic nodules ↓ Tumor accumulation ↑ Immune activation ↑	[137]
Pseudoneutrophil cytokine sponges (pCSs) (Figure 4C)	in vitro: B16F10 and 4T1 cells in vivo: B16F10 and 4T1 syngeneic mice models	in vitro: 3.12–200 µg/mL in vivo (iv or it): 20 µg per mouse per injection	MDSC expansion ↓ MDSC tumor trafficking ↓ Tumor-infiltrating T lymphocytes ↑ Antitumor T cell function ↑ Tumor growth ↓ Animal survival ↑ Synergistic effect with PD-1 blockade	[138]
Neutrophil-mimicking nanodevice (FKPN) (Figure 4D)	in vitro: MDA-MB-231, 4T1, MCF-10A and HC11 cells in vivo: 4T1-xenograft in mice	in vitro: 20 µg/mL porphyrin in vivo (iv): 10 mg/kg	DNA damage ↑ Histone H1 translocation ↑ Apoptosis ↑ in situ ^1^O_2_ generation in the nucleus ↑ Tumor growth ↓ Abscopal effect ↑ DC maturation ↑ T-cell infiltration ↑ GSH depletion ↑	[139]
Nm@MSNs-DOX/SM nanocomplex (Figure 4E)	in vitro: SU-DHL-2 cells in vivo: SU-DHL-2 xenograft in nude mice	in vitro: DOX 2 µmol/L, SM 40 µmol/L in vivo (iv): DOX 2 mg/kg, SM 40 mg/kg	ROS ↑ Mitochondrial dysfunction ↑ Apoptosis ↑ Tumor growth ↓ Inflammatory cytokines (TNF-α, IL-1β) ↓ Tumor accumulation ↑ Bcl-2 ↓, Bax ↑	[140]
Neutrophil membrane-coated nanoparticles (TNM-PN) (Figure 4F)	in vitro: SKOV3, RAW264.7, HUVECs cells in vivo: SKOV3 tumor-bearing nude mice	in vitro: PTX (0.5–10 µg/mL), TRAIL (0.04–0.64 µg/mL) in vivo (iv): PTX (5 mg/kg), TRAIL (0.32 mg/kg)	TRAIL-mediated endocytosis ↑ Adhesion to inflamed endothelial cells ↑ Tumor accumulation ↑ Tumor growth ↓ Apoptosis ↑ Survival rate ↑	[141]
Neutrophil membrane-coated PLGA nanoparticles (NM-PN) (Figure 4G)		in vitro: PTX (0.5–10 µg/mL) in vivo (iv): PTX (5 mg/kg)	Adhesion to inflamed endothelial cells ↑ Tumor accumulation ↑ Tumor growth ↓ Apoptosis ↑	
NM-HB NPs-mediated PDT	in vitro: LO2 and HepG2 cells in vivo: HCC tumor-bearing mice	in vitro: 20–100 μg/mL in vivo (iv): 2 mg/mL (100 μL) + Laser stimulation (0.8 W/cm^2^ for 10 min)	ROS ↑ Apoptosis ↑ (viaCaspase-3, -7, -9 activation) JUNB expression ↓ Mitochondrial dysfunction ↑ Tumor volume ↓ TNF-α and IL-6 levels ↓	[142]
Neutrophil membrane-camouflaging nanoparticles (TNM-PN)	in vitro: SKOV3 ovarian cancer cells in vivo: SKOV3 tumor-bearing mice	in vitro: PTX (0.5–10 µg/mL), TRAIL (0.04–0.64 µg/mL) in vivo (iv): Multiple doses over 7 injections	Enhanced cellular internalization via TRAIL receptors Apoptosis ↑ Bcl-2 ↓, Bax ↑ Tumor growth ↓ Survival rate ↑ Circulation time ↑ Tumor accumulation ↑	[143]
Neutrophil membrane-coated immunomagnetic nanoparticles (Neu-IMNs)	in vitro: MCF-7 and HeLa cells in vivo: breast cancer patient samples	in vitro: 100 µg Neu-IMNs/mL in vivo: 100 µg Neu-IMNs/mL blood	Enhanced isolation and purity of CTCs Reduction in nonspecific protein adsorption Improved cell viability High capture efficiency and gene analysis viability	[144]
Urease micromotor-powered neutrophils (UM-NEs) nanodrug delivery system (Figure 5A)	in vitro: cytokine-enriched inflammatory models in vivo: carotid and lower extremity arterial thrombosis models in mice	in vitro: 100 µM urea in vivo (iv): 1 mg/kg body weight of UM-NEs (Ag-UK)	Thrombolysis efficiency ↑ (vascular recanalization restored) Hemorrhagic side effects ↓ Rethrombosis formation ↓ NETs at the thrombus site ↑ Ag-UK release triggers thrombolytic activity	[145]
Paclitaxel-loaded liposomal neutrophils (PTX-CL/NEs) (Figure 5B)	In vitro: murine brain microvascular endothelial cells, G422 tumor spheroid model In vivo: mouse glioma resection model (G422 and C6 cells)	In vitro: 50 µg PTX/mL In vivo (iv): 5 × 10⁶ PTX-CL/NEs/mouse (equivalent to 5 mg PTX/kg)	Tumor recurrence ↓ Survival rate ↑ (50% survival extended to 61 days) Brain tumor targeting ↑ (AUCbrain ~1162× higher than taxol) Drug release induced by inflammatory cytokines ↑ (NETs activation) Drug accumulation in residual glioma cells ↑	[146]
Sialic acid-modified liposomal epirubicin (EPI-SL)	in vitro: S180 tumor cells in vivo: murine S180 tumor xenograft mice model	in vitro: 50 µg/mL in vivo (iv): 5 mg/kg, 5 doses every 3 days	Tumor growth ↓ (complete tumor eradication in 50% of treated mice) Survival rate ↑ (median survival: 112 days) Intratumoral drug retention ↑ Systemic toxicity ↓ Anti-inflammatory macrophage ↓	[147]
CTX-NPs@NEs (cabazitaxel-loaded nanoparticles with neutrophils) (Figure 5C)	in vitro: 4T1 Luc breast cancer cells in vivo: bone metastasis tumor model in BALB/c mice	in vitro: 10 µg CTX/mL in vivo (iv): 1.1 mg/kg CTX equivalent	Tumor growth ↓ Bone metastases ↓ Survival rate ↑ Drug accumulation in bone marrow ↑ Bone mineral density (BMD) ↑ Splenomegaly ↓	[148]
Abraxane/human neutrophils cytopharmaceuticals (Figure 5D)	in vitro: SNU-719 cells in vivo: SNU-719 tumor-bearing mice	in vitro: 8 µg/mL of PTX released from Abraxane/NEs in vivo (iv): 2.7 mg/kg PTX (from Abraxane/NEs	Tumor growth ↓ Cytotoxicity ↑ NETs formation ↑ Inflammatory cytokines (e.g., IL-8, TNF-α) ↑ Tumor accumulation ↑	[149]
Neotype neutrophil cytopharmaceutical (NEs@STING-Mal-NP) with liposomal STING agonists (Figure 5E)	in vitro: HUVEC and 4T1 cells in vivo: 4T1 tumor-bearing mice	in vitro: 10 μg/mL (NEs@STING-Mal-NP) in vivo (iv): 3 mg/kg (NEs@STING-Mal-NP)	Macrophages, dendritic cells, and CD8+ T cells, into tumors ↑ Tumor penetration ↓ Activation of the STING pathway Intra tumor STING agonists ↑ Accumulation in tumor↑ Safety profile ↑ Cytotoxic effects ↑	[150]
Pyropheophorbide-a loaded albumin NPs (Ppa-loaded BSA NPs)	in vivo: mouse melanoma model (B16)	in vivo (iv): TA99 antibody: 40 mg/kg in vivo (iv): Cy5-BSA NPs: 8 mg/kg in vivo (iv): Ppa-loaded BSA NPs: 2 mg/kg	Neutrophil recruitment↑ Nanoparticle tumor accumulation ↑ Photodynamic therapy efficacy ↑ Tumor growth suppression ↑ Mouse survival ↑ Drug delivery to tumors ↑	[151]
Neutrophil-based delivery system for Au nanorods (AuNR) (Figure 5F)	in vitro: Lewis lung adenocarcinoma cells in vivo: Lewis tumor-bearing mice	in vitro: 10, 20, 40, 60, 80 µg/mL AuNRBR/N in vivo (iv): 100 µg/mL AuNRBR/N	Tumor targeting ↑ Tumor penetration ↑ Photothermal effect ↑ Tumor growth ↓ Survival rate ↑ Neutrophil recruitment↑ Extracellular trap release ↑	[152]
Photoactive neutrophils (PAN) encapsulated multifunctional nanocomplex (RA/Ce6) of RGD apoptotic peptide conjugate (RA) decorated Liposomal photosensitizer Ce6 (Figure 5G)	in vitro: B16F10, Cal-27, L02 and fibroblast cells in vivo: B16F10 xenograft and oral cancer model	in vitro: IC_50_: 1.45 μM (Ce6) in vivo (iv): 5 mg/kg (Ce6)	Drug felivery ↑ Mitochondrial targeting↑ Mitochondrial membrane potential disruption ↑ Cytotoxicity to cancer cells (B16F10 and Cal-27) ↑ Cytotoxicity to healthy cells (L02 and fibroblast) ↓ ROS generation ↑ Apoptosis ↑ Tumor targeting and accumulation ↑ Survival rate ↑	[153]
Inflammation-activatable engineered neutrophils: neutrophil/Dox-loaded MMSNs (ND-MMSNs) (Figure 5H)	in vitro: U87, C6 and bEnd.3 cells in vivo: U87 glioma-bearing mice	in vitro: ND-MMSNs at varied Dox concentrations (0.625, 1.25, 2.5, 5, 10, 20, and 40 μg/mL) in vivo (iv): DN-MMSNs (1×10^6^ cells/mouse, 5 mg/kg Dox)	Drug accumulation in tumor sites ↑ Cytotoxicity ↑ Apoptosis ↑ Survival rate ↑	[154]
Nanoparticle–neutrophil composites (NSNP@Ne) (Figure 5I)	in vitro: Pan02 cells in vivo: Pan02 tumor-bearing mice	in vitro: 0–200 µg/mL NSNP in vivo (iv): 10 mg/kg NSNP and 5 × 10^5^ neutrophils/kg	Photothermal cytotoxicity ↑ Tumor temperature ↑ (up to 60 °C) under NIR irradiation Tumor growth ↓ Recurrence ↓ Necrosis ↑	[155]
Nanoengineered neutrophils (Acouscyte/O₂)	in vitro: B16F10, L02 cells In vivo: B16F10 tumor-bearing mice	in vitro: 100 µg/mL (temoporfin equivalent) in vivo (iv): Temoporfin: 0.5 mg/kg	Oxygen release ↑ ROS generation ↑ Singlet oxygen (^1^O₂) production ↑ Tumor accumulation ↑ Tumor growth ↓ Survival time ↑	[156]

**↑**: Increase, **↓**: Decrease; iv: Intravenous, it: Intratumoral; 3T3: Murine fibroblast cell line; 4T1: Murine mammary carcinoma cell line; EPI-SL: Liposomal epirubicin modified with sialic acid; AuNR: Gold nanorods; B16F10: Murine melanoma cell line; bEnd.3 cells: Mouse brain endothelial cells; C6: Murine glioma cell line; Cal-27: Human oral squamous cell carcinoma cell line; Ce6: Chlorin e6; CPO: Chloroperoxidase; CTC: Circulating tumor cells; CTX-NPs@NEs: Cabazitaxel-loaded nanoparticles with neutrophils; DC: Dendritic cells; DOX: Doxorubicin; FKPN: Neutrophil-mimicking nanodevice; GCZM: Artificial “super neutrophils”; G422: Murine glioma model; GOx: Glucose oxidase; GSH: Glutathione; HC11: Mouse mammary epithelial cell line; HCC: Hepatocellular carcinoma; HeLa: Human cervical cancer cell line; HepG2: Human hepatocellular carcinoma cell line; HL-7702: Normal human liver cell line; hNVs: Hybrid cellular membrane nanovesicles; HUVECs: Human umbilical vein endothelial cells; IC_50_: 50% inhibitory concentration; ICG: Indocyanine green; L02: Human normal liver cell line; LO2: Human normal liver cell line; MCF-10A: Michigan Cancer Foundation-10A; MCF-7: Human breast cancer cell line; MDA-MB-231: Human triple-negative breast cancer cell line; MMSNs: Mesoporous silica nanoparticles; NEs@STING-Mal-NP: Neotype neutrophil cytopharmaceutical; NETs: Neutrophil extracellular traps; Neu-IMNs: Neutrophil membrane-coated immunomagnetic nanoparticles; NM-HB NPs: Neutrophil membrane hybrid biomimetic nanoparticles; NM-PN: Neutrophil membrane-coated PLGA nanoparticles; Nm@MSNs-DOX/SM: Neutrophil-membrane-coated mesoporous silica nanoparticles loaded with DOX and SM; Pan02: Mouse pancreatic adenocarcinoma cell line; pCSs: Pseudoneutrophil cytokine sponges; PNMAuDIs: Platelet–neutrophil hybrid membrane-coated gold nanocages; PTX-CL/NEs: Paclitaxel-loaded liposomal neutrophils; RAW264.7: Murine macrophage cell line; S180: Sarcoma 180; SCNG: Supramolecular core–shell nanogel system; SIRPα: Signal regulatory protein alpha; SKOV3: Human ovarian cancer cell line; SNU-719: Human Epstein–Barr virus-associated gastric carcinoma cell line; SU-DHL-2: Human diffuse large B-cell lymphoma cell line; TNM-PN: Neutrophil membrane-coated nanoparticles; TRAIL: TNF-related apoptosis-inducing ligand; U87: Human glioblastoma cell line; UM-NEs: Urease micromotor-powered neutrophils.

## Data Availability

Not applicable.

## Abbreviations

The following abbreviations are used in this manuscript:

5FU	5-Fluorouracil
Acouscyte/O₂	Nanoengineered Neutrophils as a Cellular Sonosensitizer
ADCC	Antibody-Dependent Cell-Mediated Cytotoxicity
ARG1	Arginase-1
ATRA	All-Trans Retinoic Acid
AuNR	Gold Nanorods
B16F10	Murine Melanoma Cell Line
Bax	Pro-apoptotic Protein
Bcl-2	Anti-apoptotic Protein
bEnd.3 cells	Mouse Brain Endothelial Cells
C6	Murine Glioma Cell Line
Cal-27	Human Oral Squamous Cell Carcinoma Cell Line
CCR5	C-C Chemokine Receptor Type 5
Ce6	Chlorin e6
COX2	Cyclooxygenase-2
CpG	Cytosine-phosphate-Guanine
CSF	Colony-Stimulating Factor
CTCs	Circulating Tumor Cells
CTLs	Cytotoxic T Lymphocytes
CTX-NPs@NEs	Cabazitaxel-Loaded Nanoparticles with Neutrophils
CXCL	Chemokine (C-X motif) Ligand
CXCR	C-X-C Chemokine Receptor
DC	Dendritic Cells
DNase	Deoxyribonuclease
DOX	Doxorubicin
ECM	Extracellular Matrix
EPI-SL	Sialic Acid-Modified Liposomal Epirubicin
FKPN	Neutrophil-Mimicking Nanodevice
GCZM	Artificial “Super Neutrophils”
G-CSF	Granulocyte Colony-Stimulating Factor
GM-CSF	Granulocyte-Macrophage Colony-Stimulating Factor
GSH	Glutathione
G422	Murine Glioma Model
HCC	Hepatocellular Carcinoma
HC11	Mouse Mammary Epithelial Cell Line
HeLa	Human Cervical Cancer Cell Line
HepG2	Human Hepatocellular Carcinoma Cell Line
HGF	Hepatocyte Growth Factor
HL-7702	Normal Human Liver Cell Line
HOCl	Hypochlorous Acid
hNVs	Hybrid Cellular Membrane Nanovesicles
HUVECs	Human Umbilical Vein Endothelial Cells
IC50	50% Inhibitory Concentration
ICB	Immune Checkpoint Blockade
ICG	Indocyanine Green
IFN-γ	Interferon-gamma
IL-1β	Interleukin-1 beta
IL-6	Interleukin-6
IL-8	Interleukin-8
IL-12	Interleukin-12
L02	Human Normal Liver Cell Line
LCN2	Lipocalin-2
MCF-10A	Michigan Cancer Foundation-10A
MCF-7	Human Breast Cancer Cell Line
MDA-MB-231	Human Triple-Negative Breast Cancer Cell Line
MMPs	Matrix Metalloproteinases
MMSNs	Mesoporous Silica Nanoparticles
MPO	Myeloperoxidase
mTOR	Mechanistic Target of Rapamycin
ND-MMSNs	Neutrophil/Dox-loaded Mesoporous Silica Nanoparticles
NEs@STING-Mal-NP	Neotype Neutrophil Cytopharmaceutical with Liposomal STING Agonists
NETs	Neutrophil Extracellular Traps
Neu-IMNs	Neutrophil Membrane-Coated Immunomagnetic Nanoparticles
NK cells	Natural Killer Cells
NM-HB NPs	Neutrophil Membrane Hybrid Biomimetic Nanoparticles
NM-PN	Neutrophil Membrane-Coated PLGA Nanoparticles
Nm@MSNs-DOX/SM	Neutrophil Membrane-Coated Mesoporous Silica Nanoparticles Loaded with Doxorubicin and SM
NSNP	Nanoparticle–Neutrophil Composites
NSNP@Ne	Nanoparticle–Neutrophil Composites
PAD4	Peptidylarginine Deiminase 4
PAN	Photoactive Neutrophils
Pan02	Mouse Pancreatic Adenocarcinoma Cell Line
PD-1	Programmed Cell Death Protein 1
PD-L1	Programmed Death-Ligand 1
PDT	Photodynamic Therapy
PI3K	Phosphoinositide 3-Kinase
PLGA	Poly(lactic-co-glycolic acid)
PMN-MDSCs	Polymorphonuclear Myeloid-Derived Suppressor Cells
Ppa-loaded BSA NPs	Pyropheophorbide-a Loaded Albumin Nanoparticles
PTX-CL/NEs	Paclitaxel-Loaded Liposomal Neutrophils
pCSs	Pseudoneutrophil Cytokine Sponges
RA	RGD Apoptotic Peptide Conjugate
RA/Ce6	RGD Apoptotic Peptide Conjugate Decorated Liposomal Photosensitizer Ce6
RAGE	Receptor for Advanced Glycation Endproducts
RAW264.7	Murine Macrophage Cell Line
RNS	Reactive Nitrogen Species
ROS	Reactive Oxygen Species
S180	Sarcoma 180
SCNG	Supramolecular Core–Shell Nanogel System
siRNA	Small Interfering RNA
SIRPα	Signal Regulatory Protein Alpha
SKOV3	Human Ovarian Cancer Cell Line
SNU-719	Human Epstein–Barr Virus-Associated Gastric Carcinoma Cell Line
STAT3	Signal Transducer and Activator of Transcription 3
STING	Stimulator of Interferon Genes
SU-DHL-2	Human Diffuse Large B-Cell Lymphoma Cell Line
TANs	Tumor-Associated Neutrophils
TGF-β	Transforming Growth Factor-beta
TLR4	Toll-Like Receptor 4
TME	Tumor Microenvironment
TNF-α	Tumor Necrosis Factor-alpha
TNM-PN	Neutrophil Membrane-Coated Nanoparticles
TRAIL	TNF-Related Apoptosis-Inducing Ligand
Tregs	Regulatory T Cells
TSP-1	Thrombospondin-1
U87	Human Glioblastoma Cell Line
UM-NEs	Urease Micromotor-Powered Neutrophils Nanodrug Delivery System
VEGF	Vascular Endothelial Growth Factor
